# Comparative genomic analysis reveals evidence of two novel *Vibrio *species closely related to *V. cholerae*

**DOI:** 10.1186/1471-2180-10-154

**Published:** 2010-05-27

**Authors:** Bradd J Haley, Christopher J Grim, Nur A Hasan, Seon-Young Choi, Jongsik Chun, Thomas S Brettin, David C Bruce, Jean F Challacombe, J Chris Detter, Cliff S Han, Anwar Huq, Rita R Colwell

**Affiliations:** 1 Maryland Pathogen Research Institute, University of Maryland, College Park, Maryland, USA; 2 University of Maryland Institute for Advanced Computer Studies, University of Maryland, College Park, Maryland, USA; 3 School of Biological Sciences and Institute of Microbiology, Seoul National University, Seoul 151-742, Republic of Korea, and International Vaccine Institute, Seoul, 151-818, Republic of Korea; 4 DOE Joint Genome Institute, Bioscience Division, Los Alamos National Laboratory, Los Alamos, NM 87545, USA; 5 Current address: Food and Drug Administration, Greenbelt, MD, USA; 6 Current address: Oak Ridge National Laboratory, Oak Ridge, TN, USA

## Abstract

**Background:**

In recent years genome sequencing has been used to characterize new bacterial species, a method of analysis available as a result of improved methodology and reduced cost. Included in a constantly expanding list of *Vibrio *species are several that have been reclassified as novel members of the *Vibrionaceae*. The description of two putative new *Vibrio *species, *Vibrio *sp. RC341 and *Vibrio *sp. RC586 for which we propose the names *V. metecus *and *V. parilis*, respectively, previously characterized as non-toxigenic environmental variants of *V. cholerae *is presented in this study.

**Results:**

Based on results of whole-genome average nucleotide identity (ANI), average amino acid identity (AAI), *rpoB *similarity, MLSA, and phylogenetic analysis, the new species are concluded to be phylogenetically closely related to *V. cholerae *and *V. mimicus*. *Vibrio *sp. RC341 and *Vibrio *sp. RC586 demonstrate features characteristic of *V. cholerae *and *V. mimicus*, respectively, on differential and selective media, but their genomes show a 12 to 15% divergence (88 to 85% ANI and 92 to 91% AAI) compared to the sequences of *V. cholerae *and *V. mimicus *genomes (ANI <95% and AAI <96% indicative of separate species). *Vibrio *sp. RC341 and *Vibrio *sp. RC586 share 2104 ORFs (59%) and 2058 ORFs (56%) with the published core genome of *V. cholerae *and 2956 (82%) and 3048 ORFs (84%) with *V. mimicus *MB-451, respectively. The novel species share 2926 ORFs with each other (81% *Vibrio *sp. RC341 and 81% *Vibrio *sp. RC586). Virulence-associated factors and genomic islands of *V. cholerae *and *V. mimicus*, including VSP-I and II, were found in these environmental *Vibrio *spp.

**Conclusions:**

Results of this analysis demonstrate these two environmental vibrios, previously characterized as variant *V. cholerae *strains, are new species which have evolved from ancestral lineages of the *V. cholerae *and *V. mimicus *clade. The presence of conserved integration loci for genomic islands as well as evidence of horizontal gene transfer between these two new species, *V. cholerae*, and *V. mimicus *suggests genomic islands and virulence factors are transferred between these species.

## Background

The genus *Vibrio *comprises a diverse group of gamma-proteobacteria autochthonous to the marine, estuarine, and freshwater environment. These bacteria play a role in nutrient cycling, degrade hydrocarbons, and can be devastating pathogens for fish, shellfish, and mammals as well as humans [1-5]. From 1981 to 2009, the number of validly described species within the genus increased from 21 to more than 100 [6,7]. The most notorious, *V. cholerae*, is the etiological agent of the severe diarrheal disease cholera, endemic in southeast Asia for at least 1,000 years and the cause of seven pandemics since 1817. Shown to be autochthonous to the aquatic environment globally, more than 200 serogroups of *V. cholerae *have been described. Epidemics of cholera are caused by *V. cholerae *O1 and O139, with *V. cholerae *non-O1/non-O139 strains associated with sporadic cholera cases and extraintestinal infections [8,9]. Cholera infections have been ascribed to the presence and expression of virulence genes, e.g., *ctxA*, *tcpA*, *tcpP*, and *toxT *[10,11], which are also harbored by toxigenic strains of *V. mimicus*, a phylogenetic near-neighbor of *V. cholerae*. Genomic analyses of *V. cholerae *and *V. mimicus *demonstrated significant similarity, suggesting horizontal exchange of virulence factors, such as CTXΦ and VPIs-1 and -2 [12]. Based on results of phylogenetic analyses reported by Thompson et al. [13], *V. cholerae *and *V. mimicus *should be assigned to separate genera, a taxonomic assignment not yet resolved.

The aims of this study were to describe the genomes of two *Vibrio *strains previously characterized as variant *V. cholerae *by culture-based and molecular methods [14,15], and compare them to closely related *Vibrio *genomes. Results of this study suggest these two strains represent novel species and demonstrate evidence of horizontal gene transfer with their near-neighbors, *V. cholerae *and *V. mimicus*. We present here the genomic characterization of two new *Vibrio *species, *Vibrio *sp. RC341 (for which we propose the name *Vibrio metecus*) and *Vibrio *sp. RC586 (for which we propose the name *Vibrio parilis*), that share a close phylogenetic and genomic relationship with *V. cholerae *and *V. mimicus*, but are distinct species, based on comparative genomics, average nucleotide identity (ANI), average amino acid identity (AAI), multi-locus sequence analysis (MLSA), and phylogenetic analysis. Also, we present results of a comparative genomic analysis of these two novel species with 22 *V. cholerae*, two *V. mimicus *and one each of *V. vulnificus *and *V. parahaemolyticus *(see Additional file 1). The new *Vibrio *species are characterized as *Vibrio *sp. RC341 and *Vibrio *sp. RC586, sharing genes and mobile genetic elements with *V. cholerae *and *V. mimicus*. These data suggest that *Vibrio *sp. RC341 and *Vibrio *sp. RC586 may act as reservoirs of mobile genetic elements, including virulence islands, for *V. cholerae *and *V. mimicus*, Horizontal gene transfer among these bacteria enables colonization of new niches in the environment, as well as conferring virulence in the human host. Descriptions of these species and definitions have been provided elsewhere [Haley et al., in preparation].

## Results and Discussion

### Strains

The two strains analyzed in this study, *Vibrio *sp. RC341 and *Vibrio *sp. RC586, were isolated from water samples from the Chesapeake Bay, MD in 1998 and 1999, respectively. *Vibrio *sp. RC341 and *Vibrio *sp. RC586 were presumptively classified as variant *V. cholerae *[14,15], based on similarity to the 16S ribosomal RNA of *V. cholerae*. *Vibrio *sp. RC341 appears as yellow *V. cholerae*-like cells and *Vibrio *sp. RC586 appears as green *V. mimicus*-like cells on TCBS agar. Both strains were typeable with *V. cholerae *antisera, *Vibrio *sp. RC586 as serogroup O133 and *Vibrio *sp. RC341 as serogroup O153 [14,15].

### General Genome Overview

The genomes of *Vibrio *sp. RC341 and *Vibrio *sp. RC586 span 28 and 16 contigs, respectively, and putatively encode 3574 and 3592 ORFs totaling 4,008,705 bp and 4,082,591 bp, respectively. *Vibrio *sp. RC341 encodes 91 RNAs, 71 of which are tRNAs. *Vibrio *sp. RC586 encodes 115 RNAs, 91 of which are tRNAs. The %GC content of each genome is ca. 46%, while the %GC content of *V. cholerae *strains is 47%. *Vibrio *sp. RC341 encodes 681 hypothetical proteins (19% of total ORFs) and *Vibrio *sp. RC586 encodes 719 hypothetical proteins (19.6% of total ORFs) determined by subsystem annotation. Twenty-four of these hypothetical proteins of *Vibrio *sp. RC586 and 48 of *Vibrio *sp. RC341 showed no homology to any of the sequences in the NCBI database.

Both genomes putatively encode two chromosomes, determined by comparing both chromosomes of *V. cholerae *N16961 to draft genome sequences of *Vibrio *sp. RC341 and *Vibrio *sp. RC586 using the MUMmer program [16] (see Additional files 2 and 3). The smaller chromosome of *Vibrio *sp. RC586 putatively encodes 1035 predicted ORFs, totaling approximately 1,155,676 bp. By this method, 951 ORFs were detected in *Vibrio *sp. RC341 totaling 987,354 bp. The smaller size of the second chromosome of *Vibrio *sp. RC341 can be attributed to low-quality coverage of this genome or uncaptured gaps. Both putative small chromosomes of the two species encode a superintegron region homologous to that of *V. cholerae*. The superintegron region of *Vibrio *sp. RC586 is ca. 93.6 kb, putatively encodes 96 ORFs, 66 (69%) of which are hypothetical proteins and the superintegron region of *Vibrio *sp. RC341 is ca. 68.6 kb, putatively encodes 66 ORFs, only 17 (26%) of which are hypothetical proteins. Interestingly, the superintegron of *Vibrio *sp. RC341 encodes several membrane bound proteins suggesting their role in the interaction with the extracellular environment.

### Genome Comparisons

**Figure 1:**
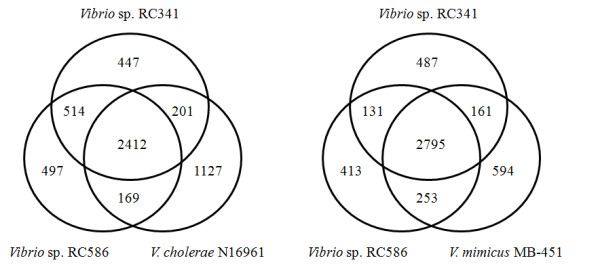
**Venn diagrams showing ORFs shared by *Vibrio *sp. RC341, *Vibrio *sp. RC586, *V. cholerae *N16961, and *V. mimicus *MB-451**. The number in the middle shows the conserved number of ORFs shared by the three strains. The numbers show that there are ORFs unique to that strain or that there are ORFs shared.

The genomes of *Vibrio *sp. RC341 and *Vibrio *sp. RC586 were compared with each other and to 22 *V. cholerae*, two *V. mimicus*, one *V. vulnificus *and one *V. parahaemolyticus *genome sequences by pairwise reciprocal BLAST analysis. *Vibrio *sp. RC341 and *Vibrio *sp. RC586 share 2104 non-duplicated ORFs (58% of the *Vibrio *sp. RC341 protein-coding genome) and 2058 non-duplicated ORFs (57% of the *Vibrio *sp. RC586 protein-coding genome) with 22 *V. cholerae *strains. Chun et al. [17] determined that the current *V. cholerae *core contains 2432 ORFs, indicating a dramatic difference in number of core genes between *Vibrio *sp. RC341/RC586 and *V. cholerae *core genomes. *Vibrio *sp. RC341 shares 2613 ORFs with *V. cholerae *N16961 (73% of *V*. sp. RC341), and *Vibrio *sp. RC586 shares 2581 ORFs with *V. cholerae *N16961 (71% of *Vibrio *sp. RC586) (Figure 1). *Vibrio *sp. RC341 shares 2956 ORFs with *V. mimicus *MB-451 (82% of *Vibrio *sp. RC341), and *Vibrio *sp. RC586 shares 3048 ORFs with *V. mimicus *MB-451 (84% of *Vibrio *sp. RC586) (Figure 1). *Vibrio *sp. RC341 and *Vibrio *sp. RC586 share 2926 ORFs with each other (81% of ORFs in both genomes) (Figure 1).

To determine average nucleotide identity (ANI) and average amino acid identity (AAI) between each genome, the average pairwise similarity between ORFs conserved between the compared genomes was calculated, following methods of Konstantinidis and Tiedje [18] and Konstantinidis et al. [19]. In this approach, two genomes with an ANI >95% and AAI >96% belong to the same species, while those with ANI and AAI below these thresholds, comprise separate species [19,20]. The ANI and AAI between *Vibrio *sp. RC586 and *Vibrio *sp. RC341 was 85 and 92%, respectively (see Additional files 4, 5, and 6). The ANIs between *Vibrio *sp. RC586 and individual *V. cholerae *ranged between 84 and 86%, while the ANI between *Vibrio *sp. RC341 and *V. cholerae *ranged between 85 and 86% (see Additional files 4, 5, and 6). The AAIs between *Vibrio *sp. RC341 and individual *V. cholerae *genomes and *Vibrio *sp. RC341 and *V. cholerae *were 92% in all comparisons (data not shown). The ANIs between *Vibrio *sp. RC586 and *V. mimicus *MB-451 and VM223 were 88% and 87%, respectively, and 86% for *Vibrio *sp. RC341 and both *V. mimicus *genomes (see Additional files 4, 5, and 6). The AAI between *Vibrio *sp. RC341 and *V. mimicus *strains MB-451 and VM223 was 92% in both comparisons, while the AAI between *Vibrio *sp. RC586 and both *V. mimicus *strains was 93% (data not shown).

The *V. cholerae *genomes had ANI >95% and AAI >96% and both *V. mimicus *strains a 98% ANI and AAI. The ANI for all *V. cholerae *and both *V. mimicus *strains was 86%. Based on these data, it is concluded that *Vibrio *sp. RC341 and *Vibrio *sp. RC586 are, indeed, separate species, genetically distinct from *V. mimicus *and *V. cholerae *and from each other. Strains of interspecies comparisons shared <95% ANI and <96% AAI with members of other species included in this study, the threshold for species demarcation [19,20], as applied to *Vibrio*, *Burkholderia*, *Escherichia*, *Salmonella*, and *Shewanella *spp. [21,19,22]. When *Vibrio *sp. RC341 and *Vibrio *sp. RC586 were compared with the more distantly related *V. vulnificus *and *V. parahaemolyticus*, *Vibrio *sp. RC586 showed 72 and 72% ANI and 73 and 73% AAI, respectively and *Vibrio *sp. RC341 73 and 72% ANI and 73 and 73% AAI with *V. vulnificus *and *V. parahaemolyticus*, respectively (see Additional files 4, 5, and 6). Furthermore, comparative analysis of the *rpoB *sequence demonstrates that *Vibrio *sp. RC341 and *Vibrio *sp. RC586 have <97.7% sequence identity with the *rpoB *sequences of all *V. cholerae *and *V. mimicus *strains included in this study. In a comparative DNA-DNA hybridization and ANI analysis, Adékambi et al. [23] demonstrated that *rpoB *<97.7% correlated with DNA-DNA hybridization <70% and ANI <95%, both being interpreted as demarcation thresholds for bacteria. All *V. cholerae *strains included in this study showed >99.5% *rpoB *sequence similarity with *V. cholerae *N16961 (data not shown). Based on a standard MLSA for the *Vibrionaceae *[21], *Vibrio *sp. RC341 and *Vibrio *sp. RC586 both have <95% pair-wise similarity with *V. cholerae*, *V. mimicus*, *V. vulnificus*, and *V. parahaemolyticus *strains. All *V. cholerae *strains and both *V. mimicus *strains used in this analysis demonstrated >95% similarity between concatenated genes of like-species (data not shown). Karlin's dissimilarity signatures were also calculated between these two genomes and the *Vibrio *genomes used in this study. *Vibrio *sp. RC586 shared >10 dissimilarity with all *V. cholerae *(11.5 to 16.2), *V. vulnificus *(19.6), and *V. parahaemolyticus *(41.6) genomes, and > 7 with both *V. mimicus *strains. *Vibrio *sp. RC341 shared >10 dissimilarity for all *V. cholerae *(10.2 to 14) except *V. cholerae *B33 (9.4) and TMA21 (9.8). *Vibrio *sp. RC341 shared >10 genome signature dissimilarity with *V. parahaemolyticus *(40.2), *V. vulnificus *(16.3), and both *V. mimicus *(>14) genomes. *Vibrio *sp RC341 and RC586 shared a genomic dissimilarity of 8.7 with each other. Taken together these data indicate that *Vibrio *sp. RC341 and *Vibrio *sp. RC586 are new species with a high genomic relatedness to *V. cholerae *and *V. mimicus*.

### Evolution of *Vibrio *sp. RC341 and *Vibrio *sp. RC586 Lineages

**Figure 2:**
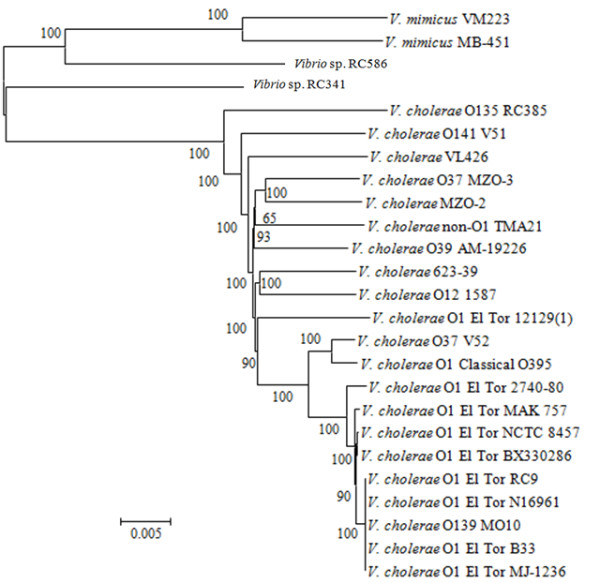
**Neighbor-joining tree based on 362,424 bp alignment of homologous sequences using the Kimura-2 parameter for nucleotide substitution**. The bootstrap supports, as percentage, are indicated at the branching points. Bar represents 0.005 substitutions per site.

The phylogenies of *Vibrio *sp. RC341 and *Vibrio *sp. RC586 were inferred by constructing a supertree, using a 362,424 bp homologous alignment of *V. cholerae*, *V. mimicus*, and the new species (Figure 2). Based on the supertree analysis *Vibrio *sp. RC341 and *Vibrio *sp. RC586 are deeply rooted in ancestral nodes, suggesting ancient evolution of the two species. Results of this phylogenetic analysis suggest the *Vibrio *sp. RC341 lineage evolved from a progenitor of the *V. cholerae *and *V. mimicus *lineages (Figure 2), a finding supported by strong bootstrap support and further evidenced by the evolutionary distance of *V. cholerae *and *V. mimicus *from *Vibrio *sp. RC341 (see Additional file 7). The two *V. mimicus *strains are interspersed among *V. cholerae*, with respect to evolutionary distance, suggesting that evolutionary distances of *V. cholerae *and *V. mimicus *are equidistant from *Vibrio *sp. RC341 (see Additional file 7).

The phylogeny of *Vibrio *sp. RC586 suggests it evolved from an ancestral member of the *V. mimicus *lineage after the lineage evolved from a progenitor of *V. mimicus*/*V. cholerae *(Figure 2). These iterations are supported by strong bootstrap support calculations. A close evolutionary relationship for *Vibrio *sp. RC586 and *V. mimicus *is also supported by shorter evolutionary distances between the *Vibrio *sp. RC586 and *V. mimicus *strains (see Additional files 8 and 9). The evolutionary distance of all genomes used in this study from *V. cholerae *BX 330286, a putative progeny of the progenitor of the 7^th ^pandemic clade [17,24], is shown in Additional file 10.

### Virulence Factors

Both *Vibrio *sp. RC586 and *Vibrio *sp. RC341 genomes encode several virulence factors found in toxigenic and non-toxigenic *V. cholerae *and *V. mimicus*. These include the *toxR*/*toxS *virulence regulators, multiple hemolysins and lipases, VSP-I and II, and a type 6 secretion system. Both VSP islands are also present in pathogenic strains of the seventh pandemic clade [25]. Although neither genome encodes CTXΦ phage, the major virulence factor encoding the cholera toxin (CT) that is responsible for the profuse secretory diarrhea caused by toxigenic *V. cholerae *and *V. mimicus*, both genomes do have homologous sequences of the chromosomal attachment site for this phage. Although these genomes do not encode TcpA, the outer membrane protein that CTXΦ attaches to during its infection cycle and ToxT, involved in CTXΦ replication and activation, they do encode several other mechanisms necessary for the complete CTXΦ life cycle and both CT production and translocation, including TolQRA, inner membrane proteins involved in CTXΦ attachment to the cell, XerCD tyrosine recombinases, which catalyze recombination between CTXΦ and the host genome, LexA, involved in CTXΦ expression, and EspD, involved in the secretion of the CTXΦ virion and CT translocation into the extracellular environment.

Neither *Vibrio *sp. RC341 nor *Vibrio *sp. RC586 encode VPI-1 or VPI-2, but *Vibrio *sp. RC341 encodes one copy of both VSP-I (VCJ_003466-VCJ_003480) and VSP-II (VCJ_000310 to VCJ_000324) and *Vibrio *sp. RC586 encodes one copy of VSP-I (VOA_002906-VOA_002918). However, neither of these strains encodes complete VSP islands, but rather variants of canonical VSP islands. Incomplete VSP islands have been frequently found in environmental *V. cholerae *and *V. mimicus *isolates [26] [Taviani et al, unpublished].

The *toxR*/*toxS *virulence regulators, hemolysins, lipases, and type 6 secretion system are present in all pathogenic and non-pathogenic strains of *V. cholerae *and both VSP islands are present in pathogenic strains of the seventh pandemic. Presence of these virulence factors in *V. cholerae *genomes sequenced to date, as well as their divergence consistent with the conserved core of *Vibrio *sp. RC341 and *Vibrio *sp. RC586, suggests that they comprise a portion of the backbone of many *Vibrio *species. Their widespread occurrence suggests the ability of all vibrios to be potential pathogens, but more likely, these factors have an important role in their ecology.

### Natural Competence

Analysis of the 22 *V. cholerae *genomes that have been sequenced revealed the presence of type IV pili genes, involved in natural transformation of *Haemophilus *spp. and *Neisseria *spp. and other competent Bacteria [27,28]. *Vibrio *sp. RC341 and *Vibrio *sp. RC586 also encode this system. Moreover, both species encode all 33 ORFs described by Meibom et al. [29,30] that comprise the chitin utilization program for induction of natural competence. The presence of these systems in the two new species and in *V. cholerae *indicates natural competence is widely employed by vibrios to incorporate novel DNA into their genomes and, thereby, enhance both adaption to new environments and in evolution. Furthermore, the well-established association of these bacteria with chitinous organisms and with high densities in biofilms [31] supports the notion that natural competence and horizontal gene transfer are both highly expressed and common in vibrios.

### Genomic Islands and Integration Loci for Exogenous DNA

Analysis of 23 complete and draft *V. cholerae *genomes by Chun et al. [17] showed 73 putative genomic islands to be present. By pairwise reciprocal comparison, the genomes of *Vibrio *sp. RC341 and *Vibrio *sp. RC586 are concluded to encode several of these genomic islands, as well as many of the insertion loci of *V. cholerae *genomic islands [17], indicating extensive horizontal transfer of genomic islands. *V. cholerae *insertion loci are not specific to individual genomic islands, but can act as integration sites for a variety of islands [17]. *Vibrio *sp. RC586 contains 33 putative GI insertion loci and *Vibrio *sp. RC341 contains 40 that are homologous to those found in *V. cholerae*. In addition to having highly similar attachment sequences and insertion loci, as found in *V. cholerae*, most of the homologous tRNA sequences between *Vibrio *sp. RC341, *Vibrio *sp. RC586, and *V. cholerae *are identical. However, three glutamine-tRNA and one aspartate-tRNA sequence of *Vibrio *sp. RC586 and four glutamine-tRNA and four aspartate-tRNA sequences of *Vibrio *sp. RC341 show between 99 and 97% similarity with homologous *V. cholerae *tRNA sequences. These sites serve as integration loci for many pathogenicity islands. Interestingly, all tRNA-Ser, the loci most commonly targeted by island encoded integrases of mobile elements in *V. cholerae *[32], were 100% similar between all strains. This high similarity of platforms serving to insert exogenous DNA suggests that the same or highly similar genomic islands are readily shared. Sequences that are characteristic of GIs and islets with homologous *V. cholerae *insertion loci and putative function and annotations are described in Additional files 11, 12, and 13.

*	Vibrio *sp. RC586 encodes eighteen sequences that are characteristic of genomic islands and islets that are also found in *V. cholerae *(see Additional file 12). Of these, VSP-I, islet-2 and GIs-2, -4, -33, -34, -35, -41, -62, -64, -73, and *Vibrio *sp. RC586-GI-1 are located on the large chromosome and islets-3 and 4, and GIs-9, -10, -20, and -61 are located on the small chromosome (see Additional file 12). The VSP-I island is located at the homologous insertion locus for VSP-I (VOA_002906-VOA_002918) in *V. cholerae *strains, but is a variant of the canonical island having a deletion in VC0175 (deoxycytidylate deaminase-related protein) and 90% sequence similarity to the canonical island.

**Figure 3:**
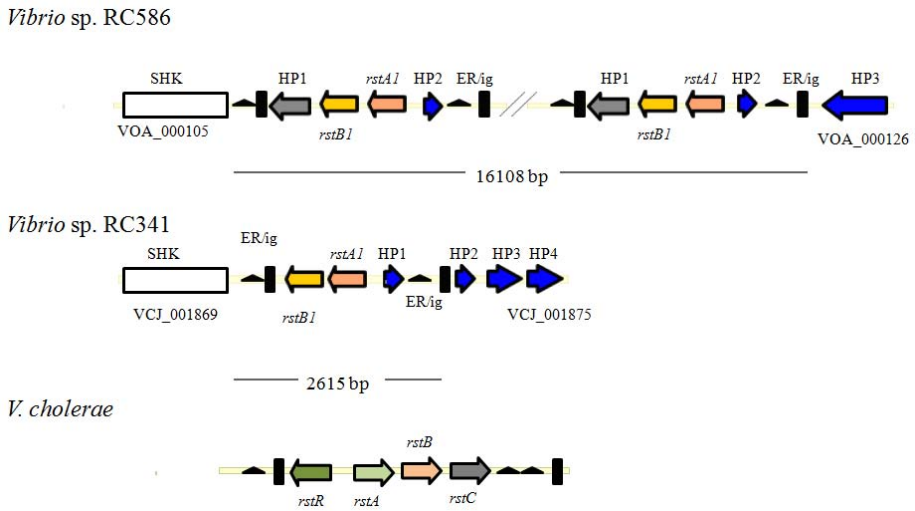
**RS1Φ-like elements located at CTXΦ attachment sites on the large chromosomes of *Vibrio *sp. RC586 and *Vibrio *sp. RC341 and the canonical RS1Φ of *V. cholerae***. SHK = sensor histidine kinase, HP = hypothetical protein, ER = end repeat, ig = intergenic region.

*Vibrio *sp. RC586 also encodes five sequences with homology to the CTXΦ attachment site, with four of them being tandemly arranged on the putative large chromosome (VOA_000105-VOA_000126). At these loci are four elements with high similarity (82 and 81% AAI) to the RS1Φ phage-like elements (*rstA1 *and *rstB1*) of *V. cholerae *SCE264 [33] and 97 to 100% nucleotide identity to the RS1Φ-like elements in *V. cholerae *TMA21, TM11079-80, VL426, and 623-39, reported by Chun et al. [17] to be GI-33 (Figure 3). RS1Φ is a satellite phage related to CTXΦ and assists in integration and replication of the CTXΦ [34,35]. However, these *V. cholerae *strains were either CTXΦ-negative or encode a CTXΦ on the other chromosome, while encoding sequences with high similarity to *rstA*, and *rstB *of RS1Φ, RS1-type sequences [33]. Immediately upstream of the *rstA1*-like sequence is an hypothetical protein and immediately downstream of this *rstB1*-like sequence is an hypothetical protein with 52% identity with that of *Colwellia psychrerythraea *34H, and a sequence with 99% similarity to an end-repeat (ER) region and an intergenic region (ig) of CTXΦ (Figure 3). This region may represent a novel phage containing ORFs with similarity to the RS1Φ satellite phage and ER and ig-1 regions with high similarity to CTXΦ. Absence of an integrase in this region suggests it may integrate into the genome via XerCD tyrosine recombinases, as does CTXΦ. All putative genomic islands shared by *V. cholerae *and *Vibrio *sp. RC586 are listed in Additional file 12.

**Figure 4:**
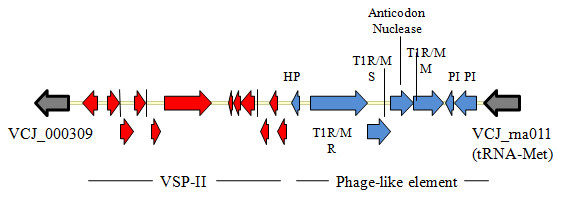
**Novel VSP-II variant found in *Vibrio *sp. RC341**. Red arrows represent VSP-II ORFs and blues arrows represent the novel phage-like region in the 3' region of the sequence. Grey arrows represent the adjacent flanking sequences. T1R/M = type I restriction modification system. PI = phage integrase.

*Vibrio *sp. RC341 putatively encodes 14 sequences that are characteristic of genomic islands and islets that are also found in *V. cholerae *(see Additional file 11). VSP-I and -II and GIs-1 to 4, 33, and islets-1 to 5 are located on the large chromosome, while GI-9 and 10 are located on the small chromosome (see Additional file 11). These GIs were described by Chun et al. [17] and two are single copies of VSP-I (VCJ_003466 to VCJ_003480) and VSP-II (VCJ_000310 to VCJ_000324). Neither of the VSP islands was present in their entirety, compared to 7^th ^pandemic *V. cholerae *strains. Similar to the VSP-I variant in *Vibrio *sp. RC586, the variant in *Vibrio *sp. RC341 has a deletion of VC0175. Also, ORFs VCJ_003468 to VCJ_003470 are annotated as phage integrase, transposase, and phage integrase, respectively. The homologous ORFs of this VSP-I variant have a 92% sequence similarity to the canonical VSP-I island. Interestingly, VSP-II variant of *Vibrio *sp. RC341 contains a 10 kb putative phage encoding a type 1 restriction modification system, has a %GC of ca. 38%, and is located at the homologous insertion locus of GI-56 in *V. cholerae *(tRNA-Met) (Figure 4). This phage shares significant similarity with *V. vulnificus *YJ016 phage (94% query coverage and 98% sequence similarity). Several variants of VSP-II are encoded in multiple strains of *V. cholerae *[E. Taviani, unpublished]. However, the variant encoded in *Vibrio *sp. RC341 is, to date, unique.

Interestingly, *Vibrio *sp. RC341 encodes *V. cholerae *GI-33, a ca. 2615 bp region, (VCJ_001870 to VCJ_001874) similar to RS1Φ-like phage in *Vibrio *sp. RC586, *V. cholerae *strains VL426, SCE264, TMA21, TM11079-80, and 623-39, showing 93 to 96% nucleotide sequence similarity across 67 to 79% of the phage (Figure 3). This region in *Vibrio *sp. RC341 encodes only the *rstA1 *and *rstB1 *and the 3' hypothetical protein flanked by CTXΦ-like end repeats and an intergenic region, inserted at the homologous CTXΦ attachment site on chromosome I (Figure 3). Analysis of this and similar phages inserting at this locus suggests an extremely high diversity of vibriophages in both structure and sequence in the environment. Putative genomic islands shared by *V. cholerae *and *Vibrio *sp. RC341 are listed in Additional file 11.

### Horizontal Gene Transfer of Genomic Islands

Homologous genomic islands typically showed higher ANI between strains than the conserved backbone regions of these genomes, an indication of recent transfer of these islands among the same and different species. All GIs shared by *Vibrio *sp. RC586 and *V. cholerae *strains were 87 to 100% ANI%, with the exception of two GIs with 77% (GI-9) and 82% (GI-62) ANI (see Additional files 12 and 13). All GIs among *Vibrio *sp. RC341 and *V. cholerae *had 87 to 99% ANI, excluding three GIs with 81 to 82% (GIs-3, 9, and 2), and two with and 85% (GI-1, *Vibrio *sp. RC341 islets -1 and -2) (see Additional files 11 and 13).

Phylogenetic analysis using homologous ORFs of the genomic islands yielded evidence of recent lateral transfer of VSP-I, and GIs-2, 41, and 61 among *V. cholerae *and *Vibrio *sp. RC586. In all cases, phylogenies inferred by the ORFs were incongruent with species phylogeny, suggesting the elements were transferred after the species diverged (see Additional files 14, 15, 16, 17, and 18). Using the same methods, we found evidence of recent lateral transfer of VSP-I, GI-4, and islet-3, between *V. cholerae *and *Vibrio *sp. RC341. In all cases, phylogenies inferred by the ORFs were incongruent with species phylogeny (see Additional files 16, 17, and 19). Our data suggests that *V. cholerae *VL426 (*V. cholerae *biotype albensis) received a VSP-I similar to that of *Vibrio *sp. RC341 and *Vibrio *sp. RC586 via horizontal gene transfer. We also found evidence of horizontal transfer of *V. cholerae *GI-2 from *V. cholerae *to *Vibrio *sp. RC586, as well as *Vibrio *sp. RC341 Islet-3 and *V. cholerae *GI-4 from *Vibrio *sp. RC341 to *V. cholerae *strains.

VSP-II, islets-2, -4, -5, and GIs-1, -2, -3, -9, -10, all present in at least one *V. cholerae *genome and in *Vibrio *sp. RC341, showed no evidence of horizontal gene transfer. Most likely there are many undescribed variants of these elements, in both structure and nucleotide sequence, yet to be found in the natural environment, with certain variants more frequently transferred among strains of the same species. Coevolution of the island and host genome over time no doubt occurs. In any case, based on the data reported here *V. cholerae *is not alone in propagating these elements. They surely cycle among different but closely related species in the environment.

### Unique Genomic Islands

*Vibrio *sp. RC586 putatively encodes five unique genomic islands and islets not yet reported for *V. cholerae *(see Additional files 12 and 13). *Vibrio *sp. RC586 GI-2 and islet-5 encode phage-like elements. Interestingly, islet-5 is annotated as probable coat protein A precursor, with similarity to bacteriophage f237 ORF5 of *V. campbellii *and zona occludens toxin (zot), with high similarity to *V. parahaemolyticus *and *V. harveyi *zot (VOA_001598-VOA_001600). This phage-like element is inserted at the homologous locus for *V. cholerae *O1 Classical CTXΦ insertion (VCA0569-VCA0570). *Vibrio *sp. RC586 GI-4 encodes sequences homologous to the Tn7 transposition *tns*ABCDE, a transposon known to integrate into phylogenetically diverse organisms and form genomic islands [36]. *Vibrio *sp. RC586 GIs-1, -3, -4, and islets-1 through 6 all share homologous insertion loci with previously described *V. cholerae *GIs (see Additional file 12).

*Vibrio *sp. RC341 encodes six putative unique genomic islands not reported before (see Additional files 11 and 13). *Vibrio *sp. RC341 GIs-1, 2, 3, 4, and 7 all encode phage-like/related elements. *Vibrio *sp. RC341 GI-4 and 7 both encode several transposases and a sequence with homology to an insertion-like sequence in the *V. parahaemolyticus *insertion sequence element ISV-3L. *Vibrio *sp. RC341 GI-6 (VCJ_002614 to VCJ002618), ca. 4962 bp region of hypothetical proteins and transposases, is inserted at the homologous locus for *V. cholerae *O1 Classical CTXΦ, a locus shown to harbor a variety of GIs and phages [17] (see Additional file 11).

## Conclusions

The genomes of two new *Vibrio *species previously characterized as variant *V. cholerae*, have been sequenced and their sequences used to describe their interesting and important features. The genomes of both species reveal significant nucleotide sequence divergence (12 to 15%) from each other and from *V. cholerae *and *V. mimicus *genomes, supporting the conclusion that both represent unique species not described before. Moreover, genes conserved among *V. cholerae*, *V. mimicus*, and the two new species varied sufficiently to suggest ancient speciation via genetic drift of the ancestral core genomic backbone. Furthermore, results of our analyses suggest *Vibrio *sp. RC341 to have evolved from a progenitor of *V. cholerae *and *V. mimicus*, whereas *Vibrio *sp. RC586 is concluded to have evolved from an early *V. mimicus *clade. Although the ANI of all genomes analyzed in this study demonstrates divergence, putative genomic islands were found to cross species boundaries, often at an higher ANI than the conserved backbone. These data, coupled with phylogenetic analyses, point to lateral transfer of the islands and phages among *V. cholerae*, *V. mimicus*, *Vibrio *sp. RC341, and *Vibrio *sp. RC586 in the natural environment. Furthermore, homologous GI insertion loci were present in both new species and in the case of *V. cholerae*, these insertion loci were not GI-specific. The pool of DNA laterally transferred between and among members of the *Vibrionaceae *strongly suggests that near-neighbors of *V. cholerae *act as reservoirs of transferable genetic elements and virulence in the environment and that *V. cholerae *is not alone in propagating these elements therein. Results of this study also demonstrate a widespread allelic variation in these elements and evidence of evolution of mobile genetic elements, including pathogenicity islands, through a multistep mosaic recombination with other elements, including phage. The ability of vibrios to incorporate exogenous DNA at several loci that encode a large combination of GIs, thereby, allows optimization of the genome for success in a specific niche or wider ecology in the natural environment.

## Methods

### Genome sequencing

Draft sequences were obtained from a blend of Sanger and 454 sequences and involved paired end Sanger sequencing on 8 kb plasmid libraries to 5× coverage, 20× coverage of 454 data, and optional paired end Sanger sequencing on 35 kb fosmid libraries to 1-2× coverage (depending on repeat complexity). To finish the genomes, a collection of custom software and targeted reaction types were used. In addition to targeted sequencing strategies, Solexa data in an untargeted strategy were used to improve low quality regions and to assist gap closure. Repeat resolution was performed using in house custom software [37]. Targeted finishing reactions included transposon bombs [38], primer walks on clones, primer walks on PCR products, and adapter PCR reactions. Gene-finding and annotation were achieved using an automated annotation server [39]. The genomes of these organisms have been deposited in the NCBI Genbank database (accession nos. NZ_ACZT00000000 and NZ_ADBD00000000).

### Comparative genomics

Genome to genome comparison was performed using three approaches, since completeness and quality of nucleotide sequences varied from strain to strain in the set examined in this study. Firstly, nucleotide sequences, as whole contigs were directly aligned using the MUMmer program [16]. Secondly, ORFs of a given pair of genomes were reciprocally compared each other, using the BLASTN, BLASTP and TBLASTX programs (ORF-dependent comparison). Thirdly, a bioinformatic pipeline was developed to identify homologous regions of a given query ORF. Initially, a segment on a target contig homologous to a query ORF was identified using the BLASTN program. This potentially homologous region was expanded in both directions by 2,000 bp, after which, nucleotide sequences of the query ORF and selected target homologous region were aligned using a pairwise global alignment algorithm [40]. The resultant matched region in the subject contig was extracted and saved as a homolog (ORF-independent comparison). Orthologs and paralogs were differentiated by reciprocal comparison. In most cases, both ORF-dependent and -independent comparisons yielded the same orthologs, though the ORF-independent method performed better for draft sequences of low quality, in which sequencing errors, albeit rare, hampered identification of correct ORFs.

To determine average nucleotide (ANI) and average amino acid identities (AAI) for the purpose of assigning genetic distances between strains and strains to species groups, a recripocal best match BLASTN analysis was performed for each genome. The average similarity between genomes was measured as the average nucleotide identity (ANI) and average amino acid identity (AAI) of all conserved protein-coding genes, following the methods of Konstantinidis and Tiedje [41]. By this method, AAI>95% and ANI>94% with >85% of protein-coding genes conserved between the pair of genomes, is judged to correspond to strains of the same species, whereas AAI<95% and ANI <94% and <85% conservation of protein-coding genes indicate different species. Dinucleotide relative abundances were determined for each genome used in this analysis. Genomic dissimilarities between genomes were determined following the methods of Karlin et al. [42]. A multi-locus sequence analysis (MLSA) was determined following standard methods for the *Vibrionaceae *[21]. Data for the MLSA were reported as percent similarity between concatenated homologous ORFs for the genomes which encoded these ORFs. These criteria were applied to results of the analyses employed in this study.

### Identification and annotation of genomic islands

Putative genomic islands (GIs) were defined as a continuous array of five or more ORFs discontinuously distributed among genomes of test strains following the methods of Chun et al [17]. Correct transfer or insertion of GIs was differentiated from deletion events by comparing genome-based phylogenetic trees and complete matrices of pairwise orthologous genes between test strains. Identified GIs were designated, and annotated using the BLASTP search of its member ORFs against the Genbank nr database. Arrays of continuous unique ORFs annotated as encoding phage-related elements and/or transposases were also identified as putative genomic islands. Genomic islets were identified as regions less than 5 ORFs and flanked by genomic island insertion loci [17]. Putative genomic islands were also investigated using the web-based application IslandViewer [43].

### Phylogenetic analyses employing genome sequences

A set of orthologues for each ORF of *V. cholerae *N16961 was obtained for different sets of strains, and individually aligned using the CLUSTALW2 program [44]. The resultant multiple alignments were concatenated to generate genome scale alignments that were subsequently used to reconstruct the neighbor-joining phylogenetic tree [45]. The evolutionary model of Kimura was used to generate the distance matrix [46]. The MEGA program was used for phylogenetic analysis [47].

## Authors' contributions

J.C., A.H. and R.R.C. designed research; T.S.B., D.C.B., J.C.D., C.S.H., N.A.H. performed research; J.C., C.J.G., N.A.H., B.J.H., and S.Y.C. analyzed data; B.J.H. and R.R.C. wrote the paper. All authors have read and approved the manuscript.

## Supplementary Material

Additional file 1 ***Vibrio *strains used in the comparative genomics utilized in this study**. Species, strain ID, serogroup/serotype and biotype (where available), geographical location and source of isolation and year of isolation are listed in this table. NCBI Genbank accession numbers are listed in the right column. Click here for file

Additional file 2 **MUMmer plot of *Vibrio *sp. RC586 as query and *V. cholerae *N16961 as reference**. *Vibrio *sp. RC586 contigs are on Y-axis and *V. cholerae *N16961 chromosomes are on X-axis. *V. cholerae *N16961 chromosome I begins at XY-intercept and chromosome II is located on the right section of the X-axis. Click here for file

Additional file 3 **MUMmer plot of *Vibrio *sp. RC341 as query and *V. cholerae *N16961 as reference**. *Vibrio *sp. RC341 contigs are on Y-axis and *V. cholerae *N16961 chromosomes are on X-axis. *V. cholerae *N16961 chromosome I begins at XY-intercept and chromosome II is located on the right section of the X-axis. Click here for file

Additional file 4 **Average nucleotide identity analysis of *Vibrio *sp. RC341**. Average nucleotide identity (ANI%) between *Vibrio *sp. RC341 and *Vibrio *genomes used in this study. Click here for file

Additional file 5 **Average nucleotide identity analysis of *Vibrio *sp. RC586**. Average nucleotide identity (ANI%) between *Vibrio *sp. RC586 and *Vibrio *genomes used in this study. Click here for file

Additional file 6 **BLAST atlas key**. BLAST atlas key for Additional files 3 and 4. Click here for file

Additional file 7 **Evolutionary distance analysis of *Vibrio *sp. RC341**. Evolutionary distance of strains used in this study from *Vibrio *sp. RC341 as determined by ANI between *Vibrio *sp. RC341 and all strains used in this study. Click here for file

Additional file 8 **Evolutionary distance analysis of *Vibrio *sp. RC586**. Evolutionary distance of strains used in this study from *Vibrio *sp. RC586 as determined by ANI between *Vibrio *sp. RC586 and all strains used in this study. Click here for file

Additional file 9 **Evolutionary distance analysis of *V. mimicus *MB451**. Evolutionary distance of *Vibrio *sp. RC586 and *Vibrio *sp. RC341 from *V. mimicus *MB451 as determined by ANI between *V. mimicus *MB451 and all strains used in this study. Click here for file

Additional file 10 **Evolutionary distance analysis of *V. cholerae *BX 330286**. Evolutionary distance of *Vibrio *sp. RC586 and *Vibrio *sp. RC341 from strains *V. cholerae *BX 330286 as determined by ANI between *V. cholerae *BX 330286 and all strains used in this study. Click here for file

Additional file 11 **Putative genomic islands of *Vibrio *sp. RC341**. Putative genomic islands of *Vibrio *sp. RC341, showing insertion loci, homologous flanking loci in *V. cholerae *N16961, %GC, other carrier strains used in this study, ANI with homologous islands, δ*, direction of transfer, islands sharing same insertion loci, and annotation. Click here for file

Additional file 12 **Putative genomic islands of *Vibrio *sp. RC586**. Putative genomic islands of *Vibrio *sp. RC586, showing insertion loci, homologous flanking loci in *V. cholerae *N16961, %GC, other carrier strains used in this study, ANI with homologous islands, δ*, direction of transfer, islands sharing same insertion loci, and annotation. Click here for file

Additional file 13 **Strain legend**. Legend for Additional files 10 and 11. Click here for file

Additional file 14 **Phylogeny of the genomic island GI-2**. Phylogeny of the genomic island GI-2 as determined by reconstructing a neighbor-joining tree using the Kimura-2 parameter as a nucleotide substitution model. Click here for file

Additional file 15 **Phylogeny of the genomic island GI-41**. Phylogeny of the genomic island GI-41 as determined by reconstructing a neighbor-joining tree using the Kimura-2 parameter as a nucleotide substitution model. Click here for file

Additional file 16 **Phylogeny of the genomic island GI-4**. Phylogeny of the genomic island GI-4 as determined by reconstructing a neighbor-joining tree using the Kimura-2 parameter as a nucleotide substitution model. Click here for file

Additional file 17 **Phylogeny of VSP-I**. Phylogeny of the genomic island VSP-I as determined by reconstructing a neighbor-joining tree using the Kimura-2 parameter as a nucleotide substitution model. Click here for file

Additional file 18 **Phylogeny of the genomic island GI-61**. Phylogeny of the genomic island GI-61 as determined by reconstructing a neighbor-joining tree using the Kimura-2 parameter as a nucleotide substitution model. Click here for file

Additional file 19 **Phylogeny of *Vibrio *sp. RC341 Islet-3**. Phylogeny of *Vibrio *sp. RC341 Islet-3 as determined by reconstructing a neighbor-joining tree using the Kimura-2 parameter as a nucleotide substitution model. Click here for file

## References

[B1] Pacha RE Kiehn ED Characterization and relatedness of marine vibrios pathogenic to fish: physiology, serology, and epidemiology Journal of Bacteriology 1969 100 3 1242 1247 5391230 10.1128/jb.100.3.1242-1247.1969 PMC250304

[B2] Kushmaro A Banin E Loya Y Stackebrandt E Rosenberg E *Vibrio shiloi *sp. nov., the causative agent of bleaching of the coral *Oculina patagonica* Int J Syst Evol Microbiol 2001 51 1383 1388 11491336 10.1099/00207713-51-4-1383

[B3] Guerinot ML West PA Lee JV Colwell RR *Vibrio diazotrophicus *sp. nov., a marine nitrogen-fixing bacterium International Journal of Systematic and Evolutionary Microbiology 1982 32 3 350 357

[B4] Hada HS West PA Lee JV Stemmler J Colwell RR *Vibrio tubiashii *sp. nov., a pathogen of bivalve mollusks International Journal of Systematic and Evolutionary Microbiology 1984 34 1 1 4

[B5] Hedlund BP Staley JT *Vibrio cyclotrophicus *sp. nov., a polycyclic aromatic hydrocarbon (PAH)-degrading marine bacterium Int J Syst Evol Microbiol 2001 51 61 66 11211274 10.1099/00207713-51-1-61

[B6] Thompson CCVA Souza RC Vasconcelos ATR Vesth T Alves N Ussery DW Iida T Thompson FL Genomic Taxonomy of the Vibrios Vibrio2009 2009 Rio de Janeiro, Brasil 10.1186/1471-2148-9-258 PMC2777879 19860885

[B7] Thompson FL Iida T Swings J Biodiversity of vibrios Microbiol Mol Biol Rev 2004 68 3 403 431 10.1128/MMBR.68.3.403-431.2004 15353563 PMC515257

[B8] Huq A Small E West P Huq M Rahman R Colwell R Ecological relationship between *Vibrio cholerae *and planktonic copepods Appl Environ Microbiol 1983 45 275 283 6337551 10.1128/aem.45.1.275-283.1983 PMC242265

[B9] Nair GB Oku Y Takeda Y Ghosh A Ghosh RK Chattopadhyay S Pal SC Kaper JB Takeda T Toxin profiles of *Vibrio cholerae *non-O1 from environmental sources in Calcutta, India Appl Environ Microbiol 1988 54 12 3180 3182 3223774 10.1128/aem.54.12.3180-3182.1988 PMC204448

[B10] Davis BR Fanning GR Madden JM Steigerwalt AG Bradford HB Jr Smith HL Jr Brenner DJ Characterization of biochemically atypical *Vibrio cholerae *strains and designation of a new pathogenic species, *Vibrio mimicus* J Clin Microbiol 1981 14 6 631 639 7037833 10.1128/jcm.14.6.631-639.1981 PMC274012

[B11] Shinoda S Nakagawa T Shi L Bi K Kanoh Y Tomochika K Miyoshi S Shimada T Distribution of virulence-associated genes in *Vibrio mimicus *isolates from clinical and environmental origins Microbiol Immunol 2004 48 7 547 551 15272201 10.1111/j.1348-0421.2004.tb03551.x

[B12] Boyd EF Moyer KE Shi L Waldor MK Infectious CTXΦ and the *Vibrio *pathogenicity island prophage in *Vibrio mimicus*: evidence for recent horizontal transfer between *V. mimicus *and *V. cholerae* Infection and Immunity 2000 68 3 1507 1513 10.1128/IAI.68.3.1507-1513.2000 10678967 PMC97308

[B13] Thompson FL Swings J Thompson FL, Austin B, Swings J Taxonomy of the Vibrios Biology of the Vibrios 2006 Washington, D.C: ASM Press 29 43

[B14] Choopun N The population structure of *Vibrio cholerae *in Chesapeake Bay PhD Thesis 2004 University of Maryland, College Park, Marine Estuarine and Environmental Science

[B15] Zo YG Phylogenomic and structural analyses of *Vibrio cholerae *populations and endemic cholera PhD Thesis 2005 University of Maryland, College Park, Marine Estuarine and Environmental Science

[B16] Kurtz S Phillippy A Delcher A Smoot M Shumway M Antonescu C Salzberg S Versatile and open software for comparing large genomes Genome biology 2004 5 2 R12 10.1186/gb-2004-5-2-r12 14759262 PMC395750

[B17] Chun J Grim CJ Hasan NA Lee JH Choi SY Haley BJ Taviani E Jeon YS Kim DW Comparative genomics reveals mechanism for short-term and long-term clonal transitions in pandemic *Vibrio cholerae* Proceedings of the National Academy of Sciences 2009 106 36 15442 15447 10.1073/pnas.0907787106 PMC2741270 19720995

[B18] Konstantinidis KT Tiedje JM Genomic insights that advance the species definition for prokaryotes Proceedings of the National Academy of Sciences 2005 102 7 2567 2572 10.1073/pnas.0409727102 PMC549018 15701695

[B19] Konstantinidis KT Ramette A Tiedje JM The bacterial species definition in the genomic era Philosophical Transactions B 2006 361 1475 1929 1940 10.1098/rstb.2006.1920 PMC1764935 17062412

[B20] Konstantinidis KT Tiedje JM Prokaryotic taxonomy and phylogeny in the genomic era: advancements and challenges ahead Current opinion in microbiology 2007 10 5 504 509 10.1016/j.mib.2007.08.006 17923431

[B21] Thompson CC Vicente ACP Souza RC Vasconcelos ATR Vesth T Alves N Ussery DW Iida T Thompson FL Genomic taxonomy of vibrios BMC Evolutionary Biology 2009 9 1 258 273 10.1186/1471-2148-9-258 19860885 PMC2777879

[B22] Vanlaere E Baldwin A Gevers D Henry D De Brandt E LiPuma JJ Mahenthiralingam E Speert DP Dowson C Vandamme P Taxon K, a complex within the *Burkholderia cepacia *complex, comprises at least two novel species, *Burkholderia contaminans *sp. nov. and *Burkholderia lata *sp. nov International Journal of Systematic and Evolutionary Microbiology 2009 59 1 102 111 10.1099/ijs.0.001123-0 19126732

[B23] Adekambi T Shinnick TM Raoult D Drancourt M Complete *rpoB *gene sequencing as a suitable supplement to DNA-DNA hybridization for bacterial species and genus delineation International Journal of Systematic and Evolutionary Microbiology 2008 58 8 1807 1814 10.1099/ijs.0.65440-0 18676461

[B24] Haley BJ Grim CJ Hasan NA Taviani E Chun J Brettin TS Bruce DC Challacombe JF Detter JC Han CS The pre-seventh pandemic *Vibrio cholerae *BX 330286 El Tor genome: evidence for the environment as a genome reservoir Environmental Microbiology Reports 2010 2 1 208 216 10.1111/j.1758-2229.2010.00141.x 23766018

[B25] Dziejman M Balon E Boyd D Fraser CM Heidelberg JF Mekalanos JJ Comparative genomic analysis of *Vibrio cholerae*: genes that correlate with cholera endemic and pandemic disease Proc Natl Acad Sci USA 2002 99 3 1556 1561 10.1073/pnas.042667999 11818571 PMC122229

[B26] Grim CJ Choi J Chun J Jeon YS Taviani E Hasan NA Haley B Huq A Colwell RR Occurrence of the *Vibrio cholerae *Seventh Pandemic VSP-I Island and a New Variant OMICS: A Journal of Integrative Biology 2010 14 1 1 7 10.1089/omi.2009.0087 20141327

[B27] Barnhart BJ Herriott RM Penetration of deoxyribonucleic acid into *Haemophilus influenzae* Biochimica et Biophysica Acta 1963 76 25 39 10.1016/0006-3002(63)90004-0 14068558

[B28] Wolfgang M Lauer P Park HS Brossay L Hebert J Koomey M PilT mutations lead to simultaneous defects in competence for natural transformation and twitching motility in piliated *Neisseria gonorrhoeae* Molecular Microbiology 1998 29 1 321 330 10.1046/j.1365-2958.1998.00935.x 9701824

[B29] Meibom KL Li XB Nielsen AT Wu CY Roseman S Schoolnik GK The *Vibrio cholerae *chitin utilization program Proc Natl Acad Sci USA 2004 101 8 2524 2529 10.1073/pnas.0308707101 14983042 PMC356983

[B30] Meibom KL Blokesch M Dolganov NA Wu CY Schoolnik GK Chitin induces natural competence in *Vibrio cholerae* Science 2005 310 5755 1824 1827 10.1126/science.1120096 16357262

[B31] Pruzzo C Vezzulli L Colwell RR Global impact of *Vibrio cholerae *interactions with chitin Environ Microbiol 2008 10 6 1400 1410 10.1111/j.1462-2920.2007.01559.x 18312392

[B32] Boyd EF Almagro-Moreno S Parent MA Genomic islands are dynamic, ancient integrative elements in bacterial evolution Trends in Microbiology 2009 17 2 47 53 10.1016/j.tim.2008.11.003 19162481

[B33] Mukhopadhyay AK Chakraborty S Takeda Y Nair GB Berg DE Characterization of VPI pathogenicity island and CTXΦ prophage in environmental strains of *Vibrio cholerae* J Bacteriol 2001 183 16 4737 4746 10.1128/JB.183.16.4737-4746.2001 11466276 PMC99527

[B34] Davis BM Waldor MK Filamentous phages linked to virulence of *Vibrio cholerae* Curr Opin Microbiol 2003 6 1 35 42 10.1016/S1369-5274(02)00005-X 12615217

[B35] Faruque SM Asadulghani Kamruzzaman M Nandi RK Ghosh AN Nair GB Mekalanos JJ Sack DA RS1 element of *Vibrio cholerae *can propagate horizontally as a filamentous phage exploiting the morphogenesis genes of CTXΦ Infect Immun 2002 70 1 163 170 10.1128/IAI.70.1.163-170.2002 11748178 PMC127613

[B36] Parks AR Peters JE Tn7 elements: Engendering diversity from chromosomes to episomes Plasmid 2009 61 1 1 14 10.1016/j.plasmid.2008.09.008 18951916 PMC2614081

[B37] Han C Chain P Finishing repetitive regions automatically with Dupfinisher 2006: Citeseer 2006 142 147

[B38] Goryshin IY Reznikoff WS Tn5 *in vitro *transposition J Biol Chem 1998 273 13 7367 7374 10.1074/jbc.273.13.7367 9516433

[B39] Aziz RK Bartels D Best AA DeJongh M Disz T Edwards RA Formsma K Gerdes S Glass EM Kubal M The RAST Server: rapid annotations using subsystems technology BMC Genomics 2008 9 1 75 90 10.1186/1471-2164-9-75 18261238 PMC2265698

[B40] Myers EW Miller W Optimal alignments in linear space Comput Appl Biosci 1988 4 1 11 17 3382986 10.1093/bioinformatics/4.1.11

[B41] Konstantinidis KT Tiedje JM Towards a Genome-Based Taxonomy for Prokaryotes J Bacteriol 2005 187 18 6258 6264 10.1128/JB.187.18.6258-6264.2005 16159757 PMC1236649

[B42] Karlin S Mrazek J Campbell AM Compositional biases of bacterial genomes and evolutionary implications Journal of Bacteriology 1997 179 12 3899 3913 9190805 10.1128/jb.179.12.3899-3913.1997 PMC179198

[B43] Langille MGI Brinkman FSL IslandViewer: an integrated interface for computational identification and visualization of genomic islands Bioinformatics 2009 25 5 664 665 10.1093/bioinformatics/btp030 19151094 PMC2647836

[B44] Larkin MA Blackshields G Brown NP Chenna R McGettigan PA McWilliam H Valentin F Wallace IM Wilm A Lopez R Clustal W and Clustal X version 2.0 Bioinformatics 2007 23 21 2947 2948 10.1093/bioinformatics/btm404 17846036

[B45] Saitou N Nei M The neighbor-joining method: a new method for reconstructing phylogenetic trees Molecular Biology and Evolution 1987 4 4 406 425 3447015 10.1093/oxfordjournals.molbev.a040454

[B46] Kimura M A simple method for estimating evolutionary rates of base substitutions through comparative studies of nucleotide sequences Journal of molecular evolution 1980 16 2 111 120 10.1007/BF01731581 7463489

[B47] Kumar S Nei M Dudley J Tamura K MEGA: A biologist-centric software for evolutionary analysis of DNA and protein sequences Brief Bioinform 2008 9 4 299 306 10.1093/bib/bbn017 18417537 PMC2562624

