# Supplementary figures and images for: Comparative genomic analysis reveals evidence of two novel Vibrio species closely related to V. cholerae
Source: BMC Microbiol. 2010 May 27;10:154. doi: 10.1186/1471-2180-10-154 (PMC2889950; doi:10.1186/1471-2180-10-154)

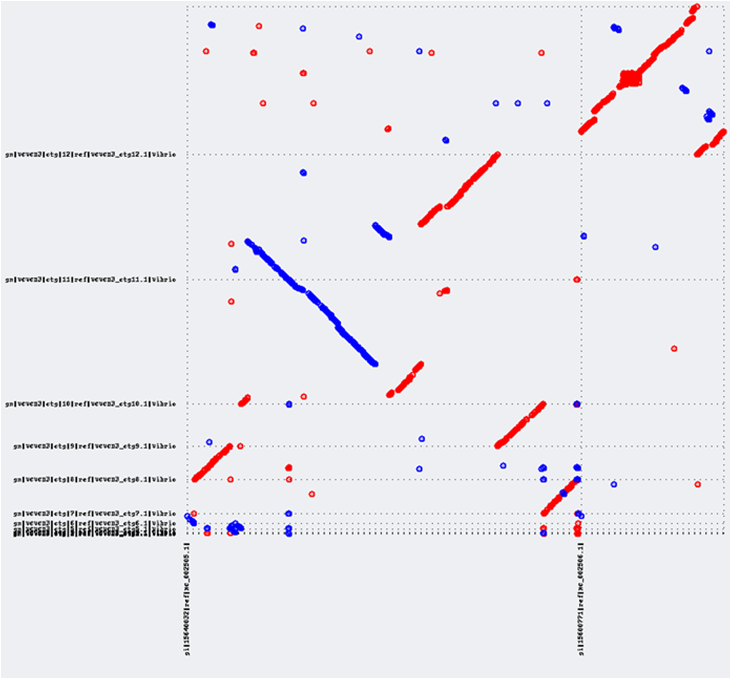

Supplement: Additional file 2 — MUMmer plot of Vibrio sp. RC586 as query and V. cholerae N16961 as reference. Vibrio sp. RC586 contigs are on Y-axis and V. cholerae N16961 chromosomes are on X-axis. V. cholerae N16961 chromosome I begins at XY-intercept and chromosome II is located on the right section of the X-axis. [file 1471-2180-10-154-S2.TIFF]

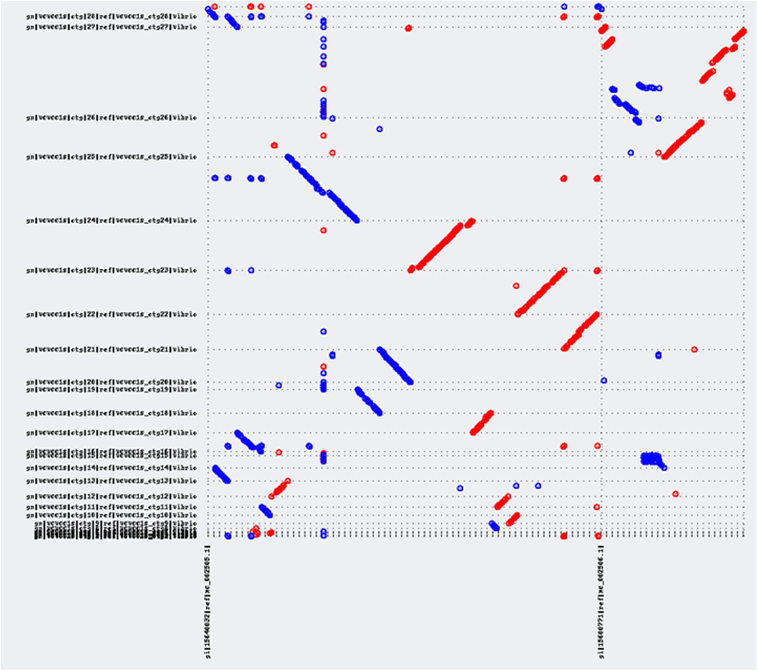

Supplement: Additional file 3 — MUMmer plot of Vibrio sp. RC341 as query and V. cholerae N16961 as reference. Vibrio sp. RC341 contigs are on Y-axis and V. cholerae N16961 chromosomes are on X-axis. V. cholerae N16961 chromosome I begins at XY-intercept and chromosome II is located on the right section of the X-axis. [file 1471-2180-10-154-S3.TIFF]

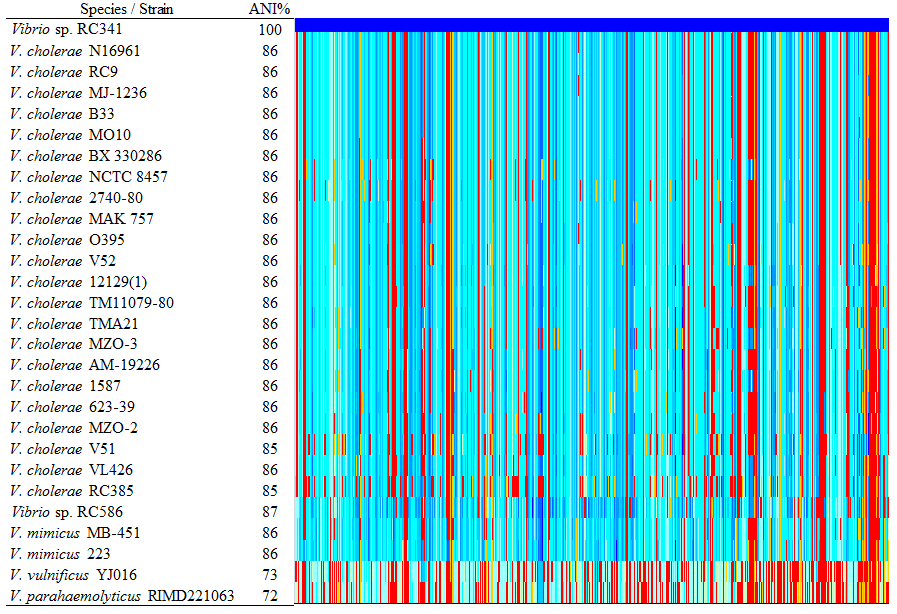

Supplement: Additional file 4 — Average nucleotide identity analysis of Vibrio sp. RC341. Average nucleotide identity (ANI%) between Vibrio sp. RC341 and Vibrio genomes used in this study. [file 1471-2180-10-154-S4.TIFF]

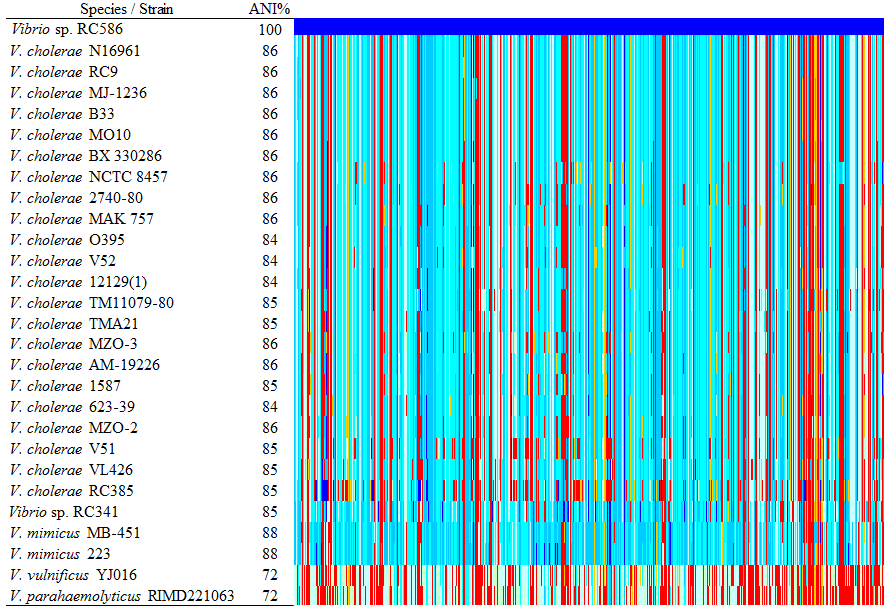

Supplement: Additional file 5 — Average nucleotide identity analysis of Vibrio sp. RC586. Average nucleotide identity (ANI%) between Vibrio sp. RC586 and Vibrio genomes used in this study. [file 1471-2180-10-154-S5.TIFF]

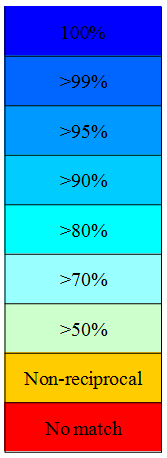

Supplement: Additional file 6 — BLAST atlas key. BLAST atlas key for Additional files 3 and 4. [file 1471-2180-10-154-S6.TIFF]

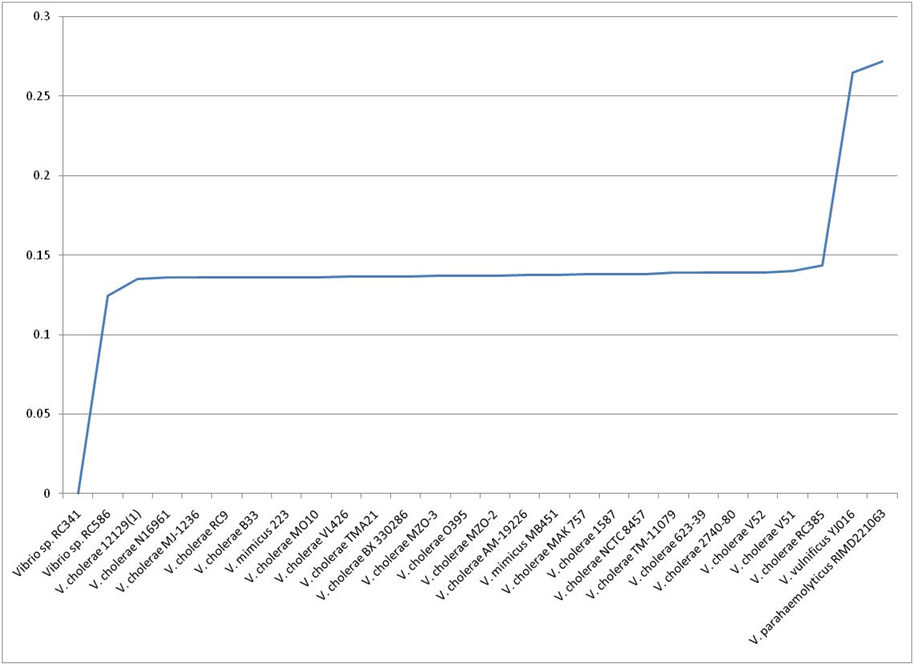

Supplement: Additional file 7 — Evolutionary distance analysis of Vibrio sp. RC341. Evolutionary distance of strains used in this study from Vibrio sp. RC341 as determined by ANI between Vibrio sp. RC341 and all strains used in this study. [file 1471-2180-10-154-S7.TIFF]

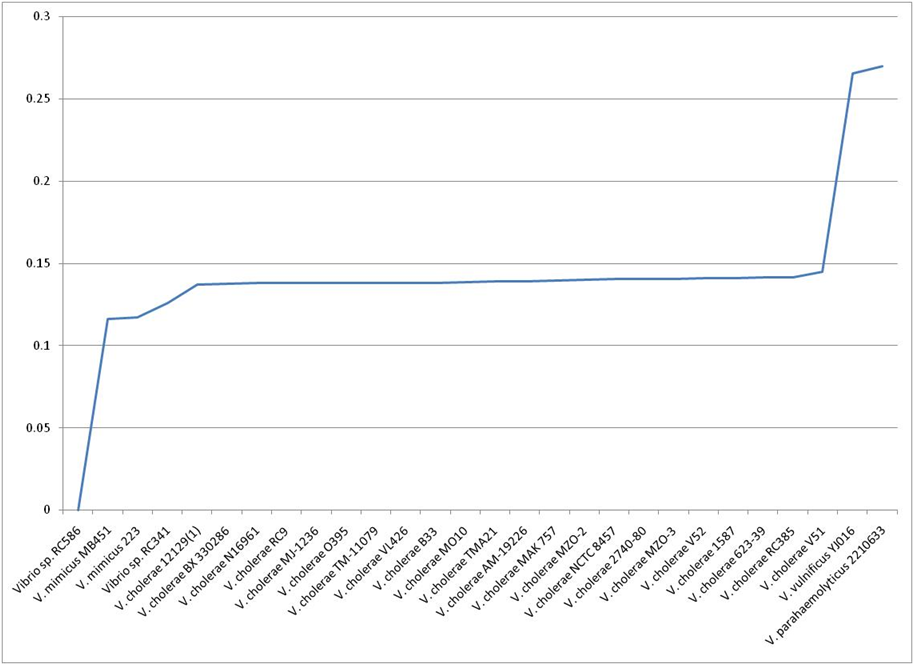

Supplement: Additional file 8 — Evolutionary distance analysis of Vibrio sp. RC586. Evolutionary distance of strains used in this study from Vibrio sp. RC586 as determined by ANI between Vibrio sp. RC586 and all strains used in this study. [file 1471-2180-10-154-S8.TIFF]

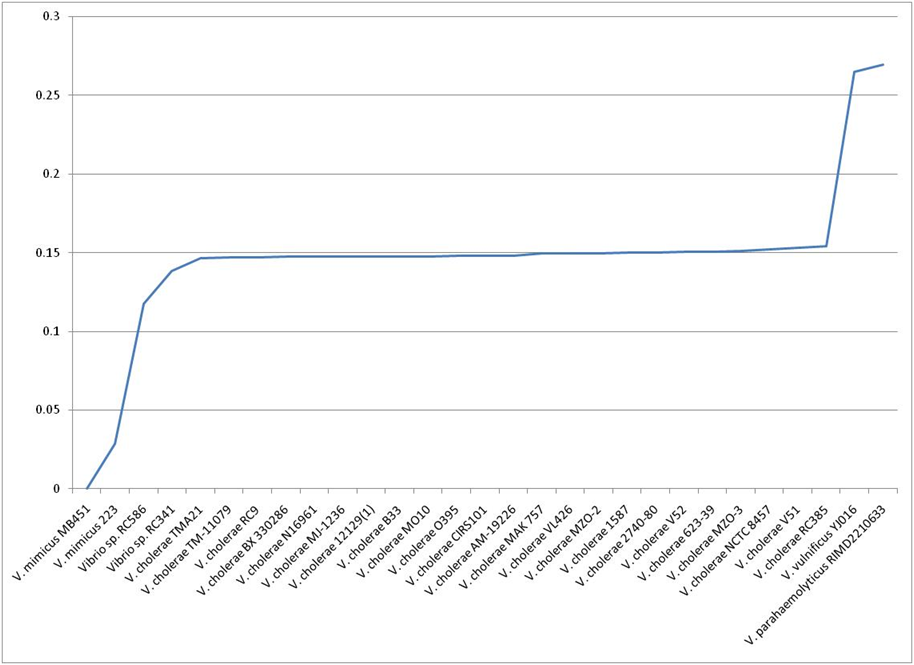

Supplement: Additional file 9 — Evolutionary distance analysis of V. mimicus MB451. Evolutionary distance of Vibrio sp. RC586 and Vibrio sp. RC341 from V. mimicus MB451 as determined by ANI between V. mimicus MB451 and all strains used in this study. [file 1471-2180-10-154-S9.TIFF]

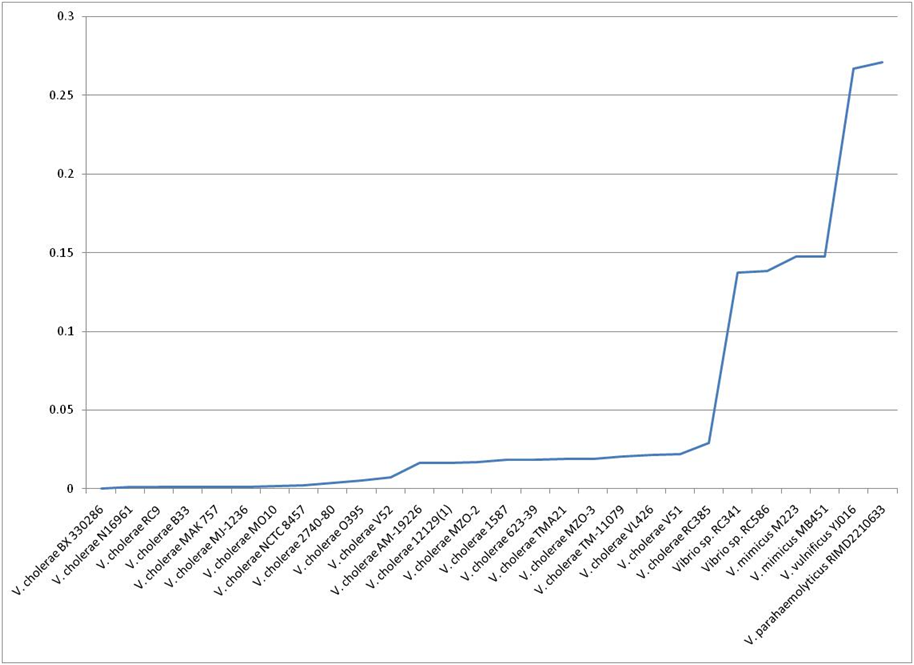

Supplement: Additional file 10 — Evolutionary distance analysis of V. cholerae BX 330286. Evolutionary distance of Vibrio sp. RC586 and Vibrio sp. RC341 from strains V. cholerae BX 330286 as determined by ANI between V. cholerae BX 330286 and all strains used in this study. [file 1471-2180-10-154-S10.TIFF]

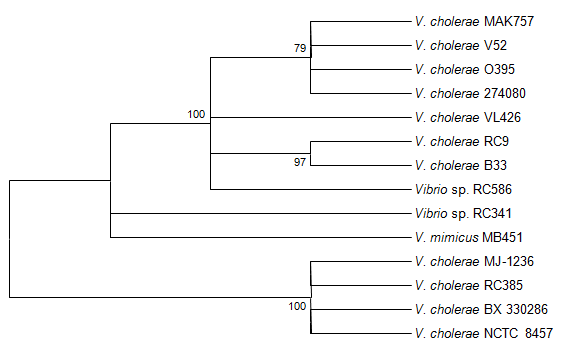

Supplement: Additional file 14 — Phylogeny of the genomic island GI-2. Phylogeny of the genomic island GI-2 as determined by reconstructing a neighbor-joining tree using the Kimura-2 parameter as a nucleotide substitution model. [file 1471-2180-10-154-S14.TIFF]

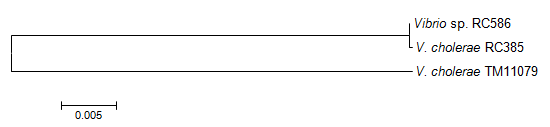

Supplement: Additional file 15 — Phylogeny of the genomic island GI-41. Phylogeny of the genomic island GI-41 as determined by reconstructing a neighbor-joining tree using the Kimura-2 parameter as a nucleotide substitution model. [file 1471-2180-10-154-S15.TIFF]

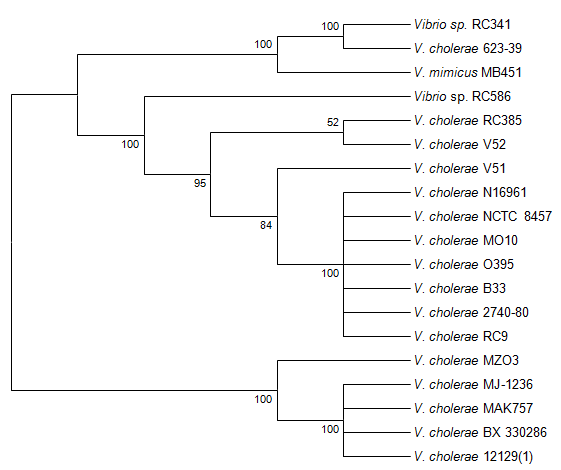

Supplement: Additional file 16 — Phylogeny of the genomic island GI-4. Phylogeny of the genomic island GI-4 as determined by reconstructing a neighbor-joining tree using the Kimura-2 parameter as a nucleotide substitution model. [file 1471-2180-10-154-S16.TIFF]

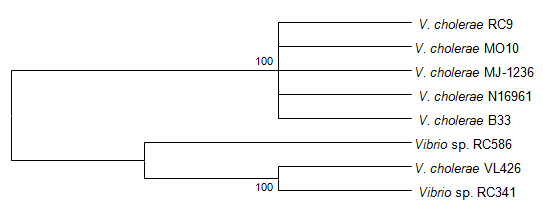

Supplement: Additional file 17 — Phylogeny of VSP-I. Phylogeny of the genomic island VSP-I as determined by reconstructing a neighbor-joining tree using the Kimura-2 parameter as a nucleotide substitution model. [file 1471-2180-10-154-S17.TIFF]

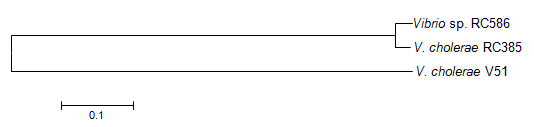

Supplement: Additional file 18 — Phylogeny of the genomic island GI-61. Phylogeny of the genomic island GI-61 as determined by reconstructing a neighbor-joining tree using the Kimura-2 parameter as a nucleotide substitution model. [file 1471-2180-10-154-S18.TIFF]

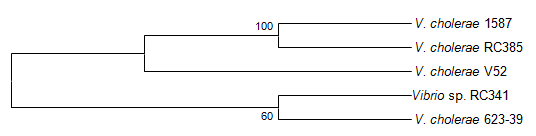

Supplement: Additional file 19 — Phylogeny of Vibrio sp. RC341 Islet-3. Phylogeny of Vibrio sp. RC341 Islet-3 as determined by reconstructing a neighbor-joining tree using the Kimura-2 parameter as a nucleotide substitution model. [file 1471-2180-10-154-S19.TIFF]
